# Nutrient intake and gender differences among Saudi children

**DOI:** 10.1017/jns.2021.95

**Published:** 2021-11-23

**Authors:** Hebah A. Kutbi

**Affiliations:** Clinical Nutrition Department, Faculty of Applied Medical Sciences, King Abdulaziz University, P.O. Box 80215, Jeddah 21589, Saudi Arabia

**Keywords:** Dietary intake, Gender differences, Macronutrients, Micronutrients, Children, Saudi Arabia

## Abstract

Dietary surveillance is necessary to determine community needs for nutrition interventions. Yet, the nutrient intake of Saudi children has not been previously investigated. The objective of the present study is to evaluate dietary data of Saudi children and investigate gender differences in nutrient intake. In this cross-sectional study, dietary data of 424 Saudi children (6–12 years of age) were collected using telephone-administered single 24-h dietary recall. Three 24-h dietary recalls were collected from a subsample of 168 children (39⋅6 %) and compared with the Dietary Recommended Intakes (DRIs). Nutrient intakes and proportions of children meeting the DRI requirements were similar and did not vary by children's gender. Over two-thirds of the children had an adequate usual intake of vitamin B12, and over half had adequate intakes (AIs) of vitamin C and phosphorus. On the other hand, our data indicated that low proportions of children consumed adequate usual intakes of magnesium and vitamin E. Over half of the children in our sample met the AI for sodium and vitamin D. Only small proportions of children met the AI for calcium, potassium and fibre. Cholesterol and saturated fat intake exceeded the limits of 300 mg and 10 % of total energy intake by 13⋅7 % (*n* 23) and 80⋅4 % (*n* 135) of the sample, respectively. Suboptimal intake of several micronutrients was observed among children, suggesting an urgent need to identify barriers to high-quality diet and to develop evidence-based interventions to promote optimal dietary efficacy for children in Saudi Arabia.

## Introduction

Dietary adequacy is crucial for children to support healthy growth and development and prevent nutrition-related diseases later in life^(1,2)^. However, existing international data indicate that many children fail to achieve dietary recommendations for multiple nutrients^(3–5)^. Thus, abundant research has been conducted to understand factors that could influence eating behaviours and dietary intake of children.

Recent studies suggest that dietary intake and preferences of children are influenced by gender, but findings in this regard were mixed. Lytle *et al.* observed similar dietary patterns for boys and girls in the USA^(6)^. In Europe, several studies reported that girls tend to consume more fruits than boys and have a stronger preference for vegetables^(7–10)^, whereas boys tend to have a greater preference for meat, processed meat, eggs and high-sugar and energy-dense foods than girls^(10)^. In a study conducted among Polish pre-schoolers, significant gender differences in multiple nutrients were observed, such as protein, saturated fat and carbohydrate^(11)^. However, research studies investigating gender differences in nutrient intakes among school-aged children are scarce.

Children are particularly vulnerable to dietary inadequacy due to higher requirements associated with physiological development and growth^(2)^. Furthermore, many children demonstrate a strong preference for some foods, while rejecting others^(12)^. In particular, school-aged children tend to demonstrate independence in selecting food choices^(13)^. These dietary behaviours may limit dietary variety and, therefore, affect dietary adequacy. In the Middle East, children's diet has been shifted from traditional dietary patterns to a Westernised diet characterised by high intakes of fast-food, energy-dense snacks and sugar-sweetened beverages^(14,15)^. Alongside, nutrient inadequacies have been documented, with suboptimal intakes of fibre, iron, zinc, calcium and vitamin D and excessive intakes of sugar, fat and saturated fat^(15)^. However, dietary intake studies in the Gulf countries, particularly in Saudi Arabia, are scarce. In fact, the need for dietary data in Saudi Arabia has been previously suggested^(16)^. Hence, the present study aimed to (1) evaluate the nutrient intake of Saudi children in relation to dietary recommendations and (2) investigate gender differences in nutrient intake. Our findings will inform policies and guide intervention programmes aimed at promoting healthy dietary habits early in life.

## Materials and methods

### Study sample

We aimed to recruit at least 176 boys and 176 girls based on the sample size calculation method suggested by Hulley *et al.*, with 95 % confidence level, 80 % power, mean energy intake of 1200 ± 350 kcal/d with a minimum of 10 % difference in energy intake between boys and girls (obtained from a pilot of 36 children which has been excluded from the total sample) and standardised effect size of 0⋅34^(17)^. Data were collected between October 2020 and February 2021. Mothers of school-aged children (6–12 years old) were invited to participate in the present study using social media channels. An online link included information on study objectives and protocol and consent for participation. The mothers were requested to answer questions on the sociodemographic characteristics of the child (age, gender, region of residence, maternal and paternal education status, maternal age and employment status, and paternal involvement in child feeding). We also asked the mothers about the appropriate date and time for communication to collect the dietary data of the child. Data of healthy Saudi children aged 6–12 years were included. The exclusion criteria include non-Saudi children and children with food allergy or any medical health condition. The final analyses included data of 424 Saudi children. The present study was conducted following the guidelines laid down in the Declaration of Helsinki. The study protocol was approved by the Faculty of Applied Medical Sciences Ethics Committee of King Abdulaziz University (FAMS-EC-2020-0010). Digital informed consent was obtained from all study participants.

### Dietary assessment

The assessment of dietary data was conducted using a single 24-h dietary recall within 2 weeks from survey data collection. A subsample of 168 children (39⋅6 %) was randomly selected to report three non-consecutive 24-h recalls (two weekdays and one weekend day). The within-person mean of the three 24-h dietary recalls was calculated and used along with the single 24-h recall data for the other children to estimate the mean intake of the total sample^(18)^. We aimed in the present study to evaluate nutrient intake from dietary food sources. Thus, we did not collect data pertaining to supplement use. A reminder text message has been sent to each mother a day before the scheduled time. During the telephone interview, mothers were educated on how to express the amount and type of food consumed by the child. We also shared pictures of serving tools to further assist in estimating the portion size of each food consumed. Mothers were requested to have the child and persons responsible for child feeding nearby and participate in the interview.

Dietary data were entered into a nutrient analysis software (Nutritics version 5.09, Dublin, Ireland) to evaluate intakes of energy, macro- and micronutrients based on Arabic foods and popular Saudi/Gulf recipes. If a food recipe was not available, information was inserted manually based on standardised recipes and later validated by two registered dietitians. Nutrient intakes of the subsample that reported three 24-dietary recalls (*n* 168) were later used to determine proportions of children who met the dietary recommendations. Given that no specific nutrient recommendations have been established for the populations in the Gulf countries, nutrient intakes of the children were compared with the U.S. and Canada's Dietary Recommended Intakes (DRIs), which have been published by the Food and Nutrition Board of the Institute of Medicine. In fact, the U.S./Canada DRIs have been widely used across the multi-ethnic populations^(19–21)^. Mean intakes of phosphorus, magnesium and vitamins E, C and B_12_ within the Estimated Average Requirements (EARs) were used to determine proportions of children with adequate nutrient intake. Mean intakes of dietary fibre, sodium, potassium, calcium and vitamin D at or above the adequate intakes (AIs) were used to determine if the children were meeting the AI^(22,23)^. Furthermore, mean intakes of cholesterol and saturated fat (>300 mg and >10 % of total energy intake, respectively) were used to identify proportions of children exceeding the recommendations of the American Academy of Pediatrics^(24)^.

### Statistical methods

Descriptive statistics were expressed as frequency (percentage), median (interquartile range) and mean ± standard deviation. The Mann–Whitney test was used to compare the intakes of nutrients for children reporting single 24-h dietary recall with the intake of children reporting three 24-h dietary recalls and to examine differences in energy and nutrient intakes by gender. The *χ*^2^ test was used to evaluate gender differences in sociodemographic characteristics and proportions of children with nutrient intake at or above the DRI requirements (EAR or AI). To evaluate gender differences in sociodemographic characteristics, *α* = 0⋅050 was used to infer significance. Bonferroni adjustments for multiple testing in dietary intake was performed; gender differences in dietary intake were determined to be significant at *α* = 0⋅003, whereas gender differences in proportions of children meeting the DRI requirements were set at *α* = 0⋅007. All statistical analyses were performed using two-sided tests carried out by the Statistical Packages for Social Sciences (SPSS) version 24.0 (Armonk, NY, USA).

## Results

### Sociodemographic characteristics of children

Approximately half of the children were boys (49⋅5 %, *n* 210). Sociodemographic characteristics of the sample are presented in Table 1. The mean age of boys and girls included in the present study were 8⋅58 ± 1⋅86 and 8⋅76 ± 1⋅84 years old, respectively. Three-quarters of the mothers (75⋅2 %, *n* 319) and two-thirds of fathers (63⋅2 %, *n* 268) had a college degree or higher. The majority of fathers were involved in child feeding (76⋅9 %, *n* 223). No significant gender difference was observed in the proportion of children by the groups of sociodemographic variables (*P* > 0⋅050).

**Table 1.** Number (proportion) of children by gender and sociodemographic characterestics^a^

Characteristic		Boys, *n* 210 (49⋅5 %)	Girls, *n* 214 (50⋅5 %)	Total, *n* 424 (100 %)	*P*-value
Age in years	6–8	108 (51⋅4)	101 (47⋅2)	209 (49⋅3)	0⋅383
	9–12	102 (48⋅6)	113 (52⋅8)	215 (50⋅7)	
Region of residence	Western	119 (56⋅7)	123 (57⋅5)	242 (57⋅1)	0⋅552
	Central	24 (11⋅4)	32 (15⋅0)	56 (13⋅2)	
	Eastern	28 (13⋅3)	25 (11⋅7)	53 (12⋅5)	
	Southern	28 (13⋅3)	20 (9⋅30)	48 (11⋅3)	
	Northern	11 (5⋅20)	14 (6⋅50)	25 (5⋅9)	
Maternal age in years	<31	35 (16⋅7)	41 (19⋅2)	76 (17⋅9)	0⋅770
	31–40	119 (56⋅7)	120 (56⋅1)	239 (56⋅4)	
	>40	56 (26⋅7)	53 (24⋅8)	109 (25⋅7)	
Maternal education	High school/diploma or less	45 (21⋅4)	60 (28⋅0)	105 (24⋅8)	0⋅115
	College degree or higher	165 (78⋅6)	154 (72⋅0)	319 (75⋅2)	
Paternal education	High school/diploma or less	81 (38⋅6)	75 (35⋅0)	156 (36⋅8)	0⋅452
	College degree or higher	129 (61⋅4)	139 (65⋅0)	268 (63⋅2)	
Maternal employment status	Unemployed	120 (57⋅1)	131 (61⋅2)	251 (59⋅2)	0⋅394
	Employed	90 (42⋅9)	83 (38⋅8)	173 (40⋅8)	
Paternal involvement in child feeding	Yes	93 (44⋅3)	108 (50⋅5)	201 (47⋅4)	0⋅405
	Sometimes	64 (30⋅5)	61 (28⋅5)	125 (29⋅5)	
	No	53 (25⋅2)	45 (21⋅0)	98 (23⋅1)	
Monthly household income in Saudi Riyal	<4000	16 (7⋅60)	13 (6⋅1)	29 (6⋅80)	0⋅882
	4000–6000	30 (14⋅3)	32 (15⋅0)	62 (14⋅6)	
	6001–10 000	51 (24⋅3)	53 (24⋅8)	104 (24⋅5)	
	10 001–15 000	50 (23⋅8)	58 (27⋅1)	108 (25⋅5)	
	>15 000	63 (30⋅0)	58 (27⋅1)	121 (28⋅5)	

a Data are presented as number (proportion).

### Dietary intake of children

**Fig. 1.**
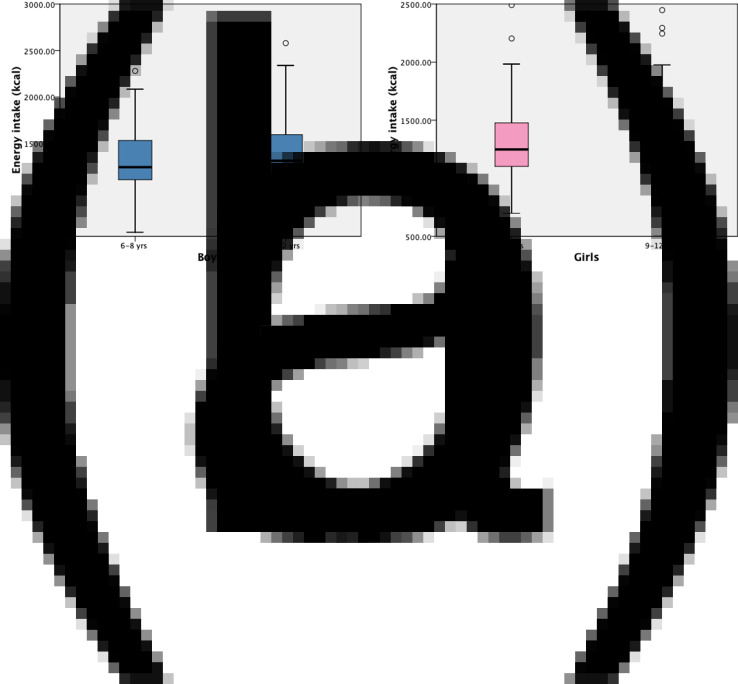
Distribution of estimated energy intake by age and gender (*n* 424): (a) estimated energy intake of boys by age; (b) estimated energy intake of girls by age.

Intakes of nutrients for children reporting single 24-h dietary recall and children reporting three 24-h dietary recalls were all similar (*P* > 0⋅050). The mean energy intake of the total sample was 1312 ± 348 kcal, with a median intake of 1247 kcal (1079–1482 kcal). Mean energy intake of boys aged 6–8 years old (1329 ± 342 kcal) did not statistically significantly differ than that of girls (1298 ± 333 kcal), *P* = 0⋅645 (Fig. 1). Similarly, the mean energy intake of boys aged 9–12 years old (1366 ± 372 kcal) did not statistically significantly differ than that of girls (1261 ± 341 kcal), *P* = 0⋅055.

**Fig. 2.**
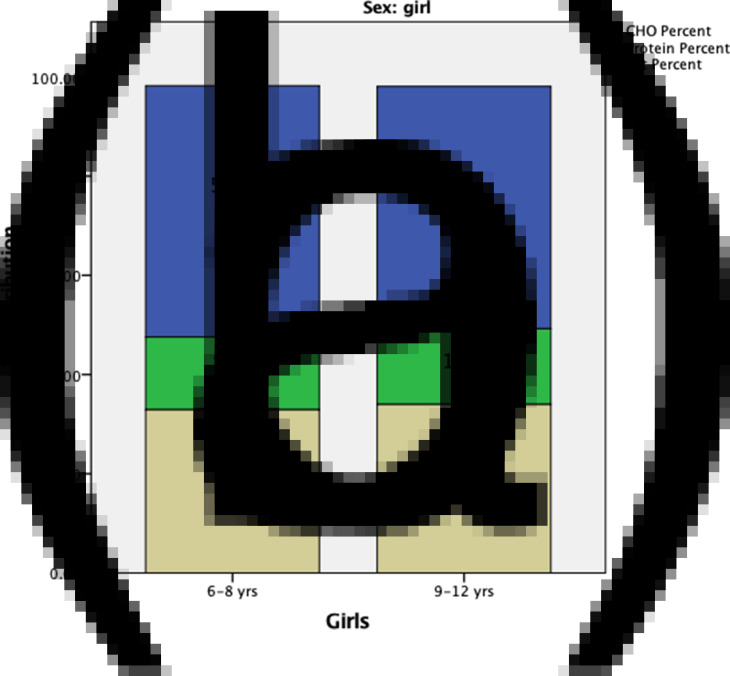
Macronutrients’ contribution to total energy intake by child gender and age (*n* 424): (a) contribution of macronutrients of boys by age; (b) contribution of macronutrients of girls by age.

Macronutrients’ contribution to total energy intake by children's age and gender are presented in Fig. 2. No significant difference was observed in the mean proportions of macronutrient intakes between boys and girls (*P* > 0⋅003), as shown in Table 2. Similarly, mean intakes of macro- and micronutrients were not found to be statistically significantly different in boys than girls (*P* > 0⋅007).

**Table 2.** Dietary intakes of children stratified by child age and gender

Dietary intake	Unit	Age group										Total (*n* 424)	
		6–8 years (*n* 209)					9–12 years (*n* 215)						
		Boys (*n* 108)		Girls (*n* 101)		*P*-value	Boys (*n* 102)		Girls (*n* 113)		*P*-value		
		Mean	Standard deviation	Mean	Standard deviation		Mean	Standard deviation	Mean	Standard deviation		Mean	Standard deviation
Protein	% Energy	15⋅4	4⋅43	14⋅7	3⋅87	0⋅319	15⋅1	3⋅71	15⋅3	4⋅12	0⋅900	15⋅1	4⋅05
	g	50⋅3	16⋅8	47⋅2	15⋅7	0⋅243	51⋅9	21⋅1	47⋅8	17⋅4	0⋅178	49⋅3	17⋅9
Carbohydrate	% Energy	48⋅8	7⋅81	50⋅7	7⋅76	0⋅149	50⋅1	45⋅7	48⋅8	6⋅96	0⋅078	49⋅6	7⋅49
	g	162	49⋅8	163	44⋅1	0⋅795	170	49⋅4	153	44⋅4	0⋅020	162	47⋅2
Fat	% Energy	33⋅7	6⋅87	32⋅9	6⋅36	0⋅546	32⋅9	6⋅64	34⋅0	6⋅54	0⋅236	33⋅4	6⋅60
	g	50⋅0	17⋅3	48⋅1	17⋅3	0⋅543	50⋅4	18⋅4	48⋅1	16⋅9	0⋅348	49⋅1	17⋅4
Cholesterol	mg	190	98⋅9	175	74⋅8	0⋅221	200	115	208	98⋅8	0⋅694	192	97⋅1
Saturated fat	g	18⋅7	6⋅70	17⋅3	4⋅55	0⋅641	18⋅0	4⋅62	18⋅7	6⋅76	0⋅224	18⋅2	5⋅71
	% Energy	12⋅4	3⋅35	12⋅5	3⋅61	0⋅894	12⋅1	3⋅71	12⋅2	3⋅68	0⋅667	12⋅3	3⋅58
Trans fat	g	0⋅30	0⋅34	0⋅30	0⋅31	0⋅599	0⋅83	4⋅77	8⋅58	10⋅0	0⋅724	6⋅16	56⋅7
Fiber	g	10⋅5	4⋅25	10⋅3	4⋅53	0⋅778	10⋅7	4⋅67	10⋅0	4⋅69	0⋅140	10⋅4	4⋅53
Sugar	g	68⋅2	31⋅7	70⋅9	27⋅6	0⋅323	68⋅2	25⋅5	65⋅4	26⋅7	0⋅567	68⋅1	28⋅0
Sodium	mg	2024	1663	2271	2087	0⋅951	2149	1410	1930	1375	0⋅049	2088	1650
Potassium	mg	1379	509	1435	504	0⋅473	1446	611	1400	617	0⋅544	1414	562
Phosphorus	mg	676	240	672	227	0⋅923	728	273	668	252	0⋅081	685	249
Magnesium	mg	141	49⋅2	139	44⋅0	0⋅894	148	61⋅8	137	56⋅9	0⋅157	141	53⋅4
Manganese	mg	2⋅27	5⋅77	1⋅64	0⋅60	0⋅820	2⋅07	2⋅74	1⋅57	0⋅82	0⋅010	1⋅89	3⋅25
Vitamin E	mg	3⋅91	3⋅32	4⋅77	5⋅07	0⋅433	4⋅46	3⋅88	4⋅14	4⋅82	0⋅194	4⋅31	4⋅33
Vitamin C	mg	54⋅7	48⋅1	57⋅3	42⋅4	0⋅469	57⋅8	57⋅2	46⋅8	48⋅5	0⋅163	54⋅0	49⋅3
Vitamin B12	μg	2⋅22	2⋅76	2⋅24	1⋅42	0⋅222	2⋅67	3⋅75	2⋅02	1⋅23	0⋅998	2⋅28	2⋅49
Iron	mg	8⋅97	3⋅04	9⋅15	3⋅01	0⋅645	9⋅57	4⋅58	9⋅07	3⋅71	0⋅934	9⋅18	3⋅61
Zinc	mg	5⋅01	2⋅09	4⋅79	1⋅95	0⋅351	5⋅08	2⋅23	4⋅78	2⋅07	0⋅363	4⋅91	2⋅08
Vitamin D	μg	23⋅9	35⋅0	15⋅4	26⋅8	0⋅488	17⋅9	33⋅8	15⋅1	29⋅4	0⋅940	18⋅7	31⋅5
Calcium	mg	572	277	581	220	0⋅411	590	246	560	255	0⋅324	575	250

Significance was set at α = 0⋅003 based on Bonferroni correction.

### Assessment of children's nutrient intakes in relation to DRI requirements

To estimate the number of children meeting the EAR or the AI requirement of each nutrient, only data of children who reported multiple dietary recalls were included (Table 3). Thus, the total sample included 168 children, wherein 50 % of the sample consisted of boys (*n* 84). No gender differences in proportions of children meeting the DRI requirements were observed (*P* > 0⋅007).

**Table 3.** Differences by gender in the number (proportion) of children meeting the DRI requirements, based on a subset of the sample (children reporting three 24-h recalls)

Dietary intake	Nutrient reference intake				Age group						Total boys (*n* 84)	Total girls (*n* 84)	*P*-value	Total sample (*n* 168)
	6–8 years		9–12 years		6–8 years (*n* 91)			9–12 years (*n* 77)						
	Boys	Girls	Boys	Girls	Boys (*n* 45)	Girls (*n* 46)	*P*-value	Boys (*n* 39)	Girls (*n* 38)	*P*-value				
Nutrients with EAR														
Phosphorus, mg	405		1055		42 (93⋅3)	46 (100)	0⋅117	4 (10⋅3)	2 (5⋅26)	0⋅675	46 (54⋅8)	48 (57⋅1)	0⋅877	94 (56⋅0)
Magnesium, mg	110		200		30 (66⋅7)	40 (87⋅0)	0⋅026	3 (7⋅69)	4 (10⋅5)	0⋅711	33 (39⋅3)	44 (52⋅4)	0⋅121	77 (45⋅8)
Vitamin E, mg	6		9		5 (11⋅1)	7 (15⋅2)	0⋅758	3 (7⋅69)	1 (2⋅63)	0⋅615	8 (9⋅52)	8 (9⋅52)	1⋅000	16 (9⋅52)
Vitamin C, mg	22		39		37 (82⋅2)	41 (89⋅1)	0⋅385	20 (51⋅3)	17 (44⋅7)	0⋅650	57 (67⋅9)	58 (69⋅0)	1⋅000	115 (68⋅5)
Vitamin B12, μg	1		1⋅5		37 (82⋅2)	43 (93⋅5)	0⋅119	27 (69⋅2)	24 (63⋅2)	0⋅635	64 (76⋅2)	67 (79⋅8)	0⋅710	131 (78⋅0)
Nutrients with AI														
Vitamin D, μg	5				28 (62⋅2)	20 (43⋅5)	0⋅090	16 (41⋅0)	21 (55⋅3)	0⋅260	44 (52⋅4)	41 (48⋅8)	0⋅758	85 (50⋅6)
Calcium, mg	800		1300		6 (13⋅3)	2 (4⋅35)	0⋅158	0 (0⋅00)	1 (2⋅63)	0⋅494	6 (7⋅14)	3 (3⋅57)	0⋅496	9 (5⋅36)
Sodium, mg	1000		1200		44 (97⋅8)	44 (95⋅7)	1⋅000	38 (97⋅4)	30 (78⋅9)	0⋅014	82 (97⋅6)	74 (88⋅1)	0⋅030	156 (92⋅9)
Potassium, mg	2300		2500	2300	2 (4⋅44)	1 (2⋅17)	0⋅617	0 (0⋅00)	2 (5⋅26)	0⋅497	2 (2⋅40)	3 (3⋅57)	0⋅014	5 (2⋅98)
Fibre, g	25		31	26	1 (2⋅22)	0 (0⋅00)	0⋅495	0 (0⋅00)	1 (2⋅63)	1⋅000	1 (1⋅19)	1 (1⋅19)	1⋅000	2 (1⋅19)

AI, adequate intake; DRI, Dietary Recommended Intake; EAR, estimated average requirement.

Significance was set at *α* = 0⋅007 based on the Bonferroni correction.

Over two-thirds of the children (78⋅0 %) had an adequate usual intake of vitamin B12 (*n* 131), and over half of the sample had AIs of vitamin C (68⋅5 %, *n* 115) and phosphorus (56⋅0 %, *n* 94). On the other hand, our data indicated that low proportions of children were having adequate usual intakes of magnesium (45⋅8 %, *n* 77) and vitamin E (9⋅52 %, *n* 16).

The majority of children in our sample (92⋅9 %, *n* 156) met the AI for sodium and 50⋅6 % met the AI for vitamin D (*n* 85). Only small proportions of children met the AI for calcium (5⋅36 %, *n* 9), potassium (2⋅98 %, *n* 5) and fibre (1⋅19 %, *n* 2). Cholesterol and saturated fat intake exceeded the limits of 300 mg and 10 % of total energy intake by 13⋅7 % (*n* 23) and 80⋅4 % (*n* 135) of the sample, respectively.

## Discussion

It is well recognised that dietary adequacy is indispensable to promote the growth and development of children. This study aimed to evaluate the nutrient intake of Saudi children and investigate gender differences in relation to dietary recommendations. Dietary intakes of boys and girls were similar. Many children had inadequate usual intakes of vitamin C and E, phosphorus and magnesium, and most children had usual intake below the AI for calcium, potassium and fibre. On the other hand, high intakes of cholesterol and saturated fat were observed. These findings suggest an urgent need to identify barriers to high-quality diet and to develop evidence-based interventions to promote optimal dietary efficacy.

Although previous studies indicated that food choices and dietary patterns might vary by child's gender^(7–10)^, our data showed similar nutrient intakes for boys and girls. According to existing data, unhealthy food choices are highly prevalent among Saudi children, demonstrated by daily consumption of sweets, sugar-sweetened beverages and energy-dense foods^(25)^. As such, it is possible that the lack of variability in nutrient intake is due to similarities in dietary habits or patterns. Further studies are needed to understand dietary behaviours of Saudi children. Specifically, future research may explore determinants of nutrient intake and food choices among Saudi children.

Across ages and genders, macronutrient distribution was consistent with the recommended ranges^(26)^. Carbohydrate was the main source of energy, followed by fat and protein. The American Academy of Pediatrics recommends that children's intake of cholesterol and saturated fat should be below 300 mg and 10 % of total energy intake, respectively^(24)^. In accordance with international data^(14,15)^, high cholesterol and saturated fat intakes were observed among children in our sample. These observations support the previously reported data that show high consumption of fast food among Saudi children^(14,15)^.

In the present study, most children had inadequate fibre intake and consumed low amount of potassium. Dietary fibre and potassium could be obtained from the consumption of whole grains, fruits and vegetables, which are often associated with high-quality diet and dietary variety^(27)^. Dietary fibre has been particularly recommended for its health promotion characteristics^(28)^. However, most Saudi children do not achieve the optimal amount of fruit and vegetables. A study conducted among 725 children in Saudi Arabia reported that 69 and 71 % of the sample were not consuming fruits and vegetables, respectively, on a daily basis, whereas only 0⋅9 % were meeting the recommendations of fruit and vegetable consumption^(29)^.

The home food environment has a paramount effect on the consumption of fruits and vegetables among children^(30)^. The limited availability and accessibility to fruits and vegetables at home can be a major determinant of low fruits and vegetable intake^(30)^. Additionally, the low price per calorie for unhealthy food options, such as sugary foods and drinks in high-income countries, may also limit the consumption of healthy food options, such as fruits and vegetables, compared to unhealthy foods^(31)^.

AIs of dietary calcium and vitamin D in children are important for normal bone mineralisation and rickets prevention^(32)^. In the present study, the mean calcium intake appeared to be very low compared to the AI for calcium. Very large proportions of school-aged children consuming an inadequate amount of calcium were also observed in several studies^(3,33,34)^. A sample of Saudi elementary school children had a mean calcium intake that represented ≤60 % of the RDA requirement and a mean vitamin D intake of approximately 23 % of the RDA^(34)^. Another study investigated the association of calcium intake with children's diet observed a significant positive strong correlation with the consumption of milk and dairy products, whereas a very weak correlation with non-dairy beverages was reported^(35)^. Further, a study by Alsubaie showed that approximately 32 % of children aged between 7 and 12 years did not consume milk or dairy products on a daily basis, whereas only 1⋅9 % were adherent to dairy intake recommendations^(29)^. On the other hand, the mean usual intake of vitamin D among children in the present study exceeded the AI, suggesting that the expected prevalence of inadequacy is low^(22)^. Nonetheless, high prevalence of vitamin D deficiency has been frequently documented. For instance, a study conducted by Mansour and Alhadidi evaluated the prevalence of vitamin D deficiency among Saudi children in Jeddah, Saudi Arabia, and reported a prevalence of 54⋅9 %^(36)^. Given that diet of the Saudi population has been frequently reported to be low in vitamin D^(34,37)^, supplementation of vitamin D may require further consideration.

Food sources of phosphorus and magnesium include a variety of protein foods, such as meats, poultry, seafood, eggs and legumes. However, the dietary data indicate inadequate intake of these nutrients among children of the older age group (9–12 years old), whereas the younger group (6–8 years old) were exceeding the EAR for dietary phosphorus and magnesium. This is most likely due to the higher requirements for the older group which make it more difficult to achieve^(38)^. Similar findings have been reported by Nasreddine *et al.*, of which large proportions of children in Saudi Arabia did not meet the respective requirements for these nutrients^(16)^.

Due to its negative impact on physical and cognitive development in children, undernutrition associated with suboptimal intake of micronutrients must be addressed^(2)^. Dietary adequacy can be achieved by consuming a balanced diet containing diverse foods, which may increase the potential to obtain a variety of nutrients^(39,40)^. Thus, intervention programmes to promote dietary variety may improve the nutritional status of children. Providing milk and dairy products and calcium-fortified foods and beverages may help children to achieve the optimal intake of calcium and phosphorus. When adequate calcium is not achieved, health professionals must consider recommending calcium supplements to maintain bone health. Additionally, fibre intake among children was found to be insufficient, whereas high intakes of saturated fat and cholesterol were observed. Interventions should focus on reducing intakes of highly processed foods and snacks, fried and fast foods, and processed meats and encourage the consumption of fibre-rich foods such as fruits and vegetables, whole grains, nuts and seeds. Children may also benefit from nutrition-based curriculum programmes to promote fruit and vegetable intakes and enhance children's diet.

The present study is one of the first to evaluate the dietary intake of Saudi children and gender differences, responding to the gap in the literature and guiding future research and intervention programmes. Even though dietary assessment has been conducted using phone interviews, studies suggest that telephone-administered dietary data produce acceptable estimates of nutrient intakes^(41)^. However, the study is limited by the convenient sampling technique, as only participants who have access to social media were recruited. Thus, our finding could be only generalisable to children of mothers with access to social media platforms. Based on current data, 96 % of the Saudi population has internet access, and 25 million Saudis are active users of social media^(42,43)^. Yet, future studies with systematically randomly sampled children are needed to determine the generalisable to all Saudi children.

## Conclusion

In summary, nutrient intakes of children were similar and did not vary by gender. The dietary data showed micronutrient inadequacies in Saudi children, with suboptimal intakes of key nutrients including fibre, calcium and phosphorus. Our findings should guide future research to further investigate barriers for optimal micronutrient intakes among Saudi children and factors. Results of the present study can serve as baseline data for fortification programmes and will inform policy-makers and other stakeholders, including funding agencies and non-governmental organisations, to address barriers to optimal nutrition and to develop culturally tailored evidence-based intervention programmes aimed at enhancing the nutritional health of Saudi children.
